# Quantitative Apparent Diffusion Coefficient Measurements Obtained by 3-Tesla MRI Are Correlated with Biomarkers of Bladder Cancer Proliferative Activity

**DOI:** 10.1371/journal.pone.0106866

**Published:** 2014-09-09

**Authors:** Sabina Sevcenco, Andrea Haitel, Lothar Ponhold, Martin Susani, Harun Fajkovic, Shahrokh F. Shariat, Manuela Hiess, Claudio Spick, Tibor Szarvas, Pascal A. T. Baltzer

**Affiliations:** 1 Department of Urology, Medical University of Vienna, Vienna, Austria; 2 Department of Pathology, Medical University of Vienna, Vienna, Austria; 3 Department of Biomedical Imaging and Image-guided therapy, Medical University of Vienna, Vienna, Austria; University of British Columbia, Canada

## Abstract

**Purpose:**

To investigate the association between Apparent Diffusion Coefficient (ADC) values and cell cycle and proliferative biomarkers (p53, p21, Ki67,) in order to establish its potential role as a noninvasive biomarker for prediction of cell cycle, proliferative activity and biological aggressiveness in bladder cancer.

**Materials and Methods:**

Patients with bladder cancer who underwent 3,0 Tesla DW-MRI of the bladder before TUR-B or radical cystectomy were eligible for this prospective IRB-approved study. Histological specimen were immunohistochemically stained for the following markers: p53, p21 and ki67. Two board-certified uropathologists reviewed the specimens blinded to DW-MRI results. Histological grade and T-stage were classified according to the WHO 2004 and the 2009 TNM classification, respectively. Nonparametric univariate and multivariate statistics including correlation, logistic regression and ROC analysis were applied.

**Results:**

Muscle invasive bladder cancer was histologically confirmed in 10 out of 41 patients. All examined tissue biomarkers were significantly correlated with ADC values (p<0.05, respectively). Based on multivariate analysis, p53 and ADC are both independent prognostic factors for muscle invasiveness of bladder cancer (>/ = T2). (p = 0.013 and p = 0.018).

**Conclusion:**

ADC values are associated with cell cycle and proliferative biomarkers and do thereby reflect invasive and proliferative potential in bladder cancer. ADC and p53 are both independent prognostic factors for muscle invasiveness in bladder cancer.

## Introduction

Bladder cancer is a malignant disease causing substantial morbidity and mortality. For optimized clinical management of patients with bladder cancer, an accurate prediction of the individual cancers biological behavior is needed. However, standard prognostic factors such as pathological staging and grading are limited in this respect [1].

Therefore, molecular biomarkers taken from tissue specimen have become increasingly investigated in order to overcome these limitations and to accurately predict tumor grade and stage [2]. Previous studies based on cell cycle and tumor proliferation markers (p53, p21, ki67) have shown a prognostic role regarding patient outcome with muscle and non-muscle bladder cancer [1], [2]. Computed tomography and magnetic resonance imaging (MRI) are regularly used for local staging of bladder cancer [3]. One of the more recent developments in MRI is the use of Diffusion-Weighted Magnetic Resonance Imaging (DW-MRI). This technique measures water diffusion by insertion of motion probing gradients in a fast T2-weighted Echo Planar Imaging sequence. A water diffusion dependent signal loss caused by spin de-phasing can be quantified by means of the Apparent Diffusion Coefficient (ADC). Recent studies have shown a promising potential of DW-MRI for detection, grading and staging in bladder cancer [4]–[6]. Microstructural changes in bladder cancers measured by ADC values correlate with the histopathological grade and stage [7], [8].

Besides these clinical prognostic factors, a recent study has shown an inverse correlation between ADC value and proliferative activity as measured by Ki67 [9]. Therefore, ADC may be described as a potential biomarker reflecting invasive and proliferative potential in bladder cancer.

Consequently, in order to follow this path of research, the aim of this study was to investigate the correlation of ADC values with cell cycle and proliferative biomarkers (p53, p21, Ki67) and to establish its potential role as a noninvasive biomarker for prediction of cell cycle, proliferative activity and biological aggressiveness in bladder cancer.

## Materials and Methods

### Patients

Patients with suspected bladder cancer that underwent 3.0 Tesla DW-MRI of the bladder before TUR-B and, in case of muscle invasive bladder cancer, subsequent radical cystectomy were eligible for this prospective study which was approved by the ethical review board of the medical university of Vienna (registration number 1749/2012). Only patients with histopathologically proven bladder cancer were included in our analysis. All patients provided written informed consent for use of anonymised data including medical images for the purpose of this study.

### MRI protocol

The examination was conducted using a whole body MRI system at a field strength of 3-Tesla (TIM Trio, Siemens, Erlangen, Germany). Dedicated vendor-supplied phased-array receiver coils were used for image acquisition. The imaging protocol included an Echo-Planar-Imaging based Diffusion Weighted Imaging (DWI) sequence (TR 7500 ms, TE_eff_ 84 ms, 3 b-values: 50, 400, 1000 s/mm^2^, parallel imaging using GRAPPA factor 2, receiver bandwidth 1736 Hz, echo spacing 0.92 ms, spatial resolution 1.8*1.5*5 mm, acquisition time 6 min.) Pixel-wise monoexponential regression of measured signal intensity values at different b-values was used to calculate Apparent Diffusion Coefficient maps.

### Histology and Immunohistochemistry

The histological specimens taken from TUR-B and, in case of muscle invasive bladder cancer, radical cystectomy were immunohistochemically stained for the following markers: p53, p21, and ki67. Two board-certified uropathologists reviewed the stained slices blinded to DW-MRI results. Further, histological grade and T-stage were classified according to the WHO 2004 and the 2009 TNM classification, respectively.

For immunohistochemical stainings on serial sections from paraffin-embedded tumor blocks BenchMark ULTRA IHC/ISH Staining Module (Ventana/Roche) with the following antibodies: p53 (Neomarkers, RM-9105-S, 1∶50 for 32 min, pretreatment ULTRA CC1-52 min), p21 (Oncogene, OP64, 1∶100 for 32 min, pretreatment ULTRA CC1-36 min), and Ki67 (Novocastra, NCL-Ki67, 1∶20 for 1 hour 12 min, pretreatment ULTRA CC1-76 min). 500 nuclei were counted in a hotspot and percentage of positive nuclei per area was evaluated within each specimen. p53 immunoreactivity was considered altered when samples demonstrated at least ≥10% nuclear reactivity [10]. p21 immunoreactivity was considered altered when samples had ≤10% staining [11]. Ki67 staining was considered to be altered when samples had >20% reactivity [12] [13] [14].

### Data analysis

Imaging data was analyzed on a dedicated workstation (Siemens Leonardo MMWP, Munich, Germany) by two independent radiologists experienced in DW-MRI and bladder cancer imaging. Solid parts of the investigated lesions were carefully identified on DWI images and ADC values were measured by placing a small (5–15 pixels) region of interest (ROI) on the ADC map avoiding postsurgical changes, necrosis or cystic tumor parts. In addition, a ROI was placed in the unaffected bladder wall. The mean ADC values were noted for further analysis. Lesion size was measured using electronic calipers on the MRI image.

### Statistical analysis

Statistical analysis was performed after testing the normal distribution of data using the Kolmogorov-Smirnoff test. ADC measurement reproducibility was addressed by calculating the coefficient of variation and the intraclass correlation coefficient. Multiple nonparametric spearman correlation analyses of averaged ADC values of both readers with immunohistochemically assessed prognostic factors and clinicopathological features were performed and the results visualized as a color-coded correlation matrix. P-values <0.05 were considered significant in this exploratory correlation analysis. Using clinically usual cut-off values for dichotomization of immunohistochemically measured biomarkers, Mann-Whitney-U tests were performed to prove group differences. For multivariate identification of independent predictors of clinicopathologic prognostic factors, binary logistic regression with forward feature selection based on likelihood ratios (entry and remove limits of 0.05 and 0.1, respectively) was performed. Nagelkerkes R-squared and the Hosmer and Lemeshow test were calculated in order to demonstrate the validity of the regression models. Predicted probabilities were saved as a variable and the area under the ROC curve (AUC) of each model was calculated using ROC analysis.

All statistical analyses were performed using the software programs R-statistics (version 3.0.3 “Warm Puppy”, the R foundation), Medcalc 13 (Medcalc, Mariakerke, Belgium) and SPSS 22 (IBM).

## Results

Forty-one patients (mean age 68y, range 41–89 years, 9 female, 31 male) were included. Of these, thirty-seven patients underwent MRI prior to TUR-B. Four patients were examined by MRI one to 27 days after TUR-B prior to cystectomy. All four patients showed bulky residual disease on MRI. There were 20 Ta, 11 T1 and 10 T2 urothelial carcinoma. Eleven patients with stage T1 received BCG therapy for one year and no patient underwent radiation or neoadjuvant chemotherapy. Median lesion size was 13 mm (IQR 19 mm) with a range of 4–80 mm. Median time between TUR-B and MRI was two days, ranging between 28 days prior to 27 days after TUR-B.

**Figure 1:**
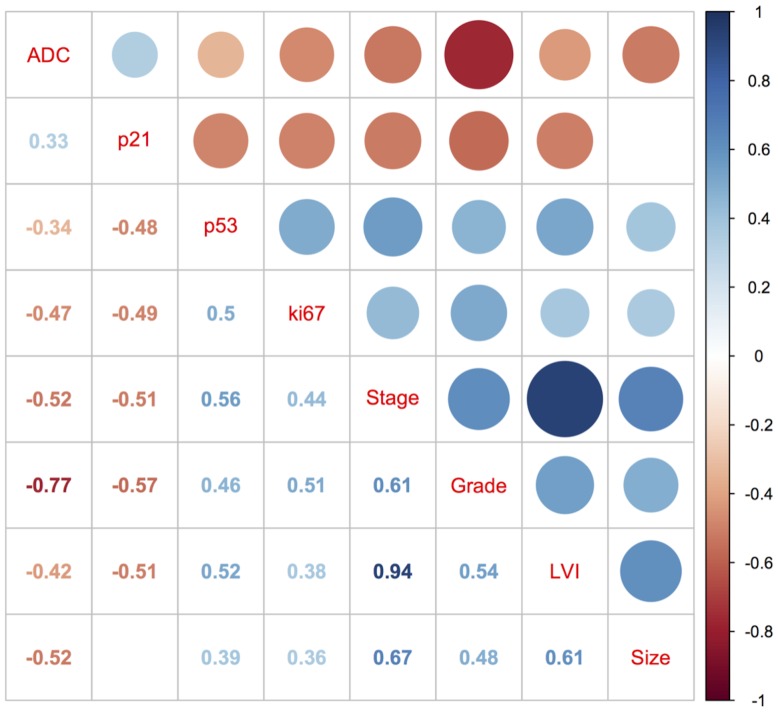
Spearman's correlation coefficient matrix with color-coded correlation coefficients (upper right denotes correlation coefficients as bubble size while lower left provides the actual coefficients as numbers). Blank spaces indicate a nonsignificant correlation defined by a P-value of >0.01.

The median bladder cancer lesion ADC value was 1.032 (IQR 0.449) *10^−3^ mm^2^/s. The coefficient of variation between both readers was 7.7%, the intraclass correlation coefficient was 0.97. The median ADC value of the unaffected bladder wall (1.338, IQR 0.384 *10^−3^ mm^2^/s) was significantly higher than the ADC value of bladder cancer (P = 0.000018). We identified significant correlations (P<0.05) between clinicopathological factors, prognostic immunohistochemical markers and ADC values obtained from DW-MRI. Regarding clinicopathological factors, ADC values were inversely correlated with tumor size (P = 0,000277), stage (P = 0,000002), lymphovascular invasion (P = 0.004) and grade (P = 6*10^−10^). Regarding molecular biomarkers, a weak positive correlation was observed between ADC and p21 (P = 0.038) and a moderate negative correlation was present between ADC and p53 (0.024) and ki67 (P = 0.007) expression. Details on correlation coefficients are given in a correlation matrix (Figure 1).

Applying clinical cut-off values for immunohistochemically derived prognostic factors, the Mann-Whitney-U test identified significant ADC group differences between p53, and ki67 (Table 1).

**Table 1:** Mean ADC values stratified by molecular biomarker results.

Prognostic factor	N	ADC (Median)	ADC (IQR)	P-value*
p21≥10%	32	1.101	0.426	0.080
p21<10%	9	0.856	0.324	
p53≥10%	24	0.906	0.489	0.030
p53<10%	17	1.205	0.254	
Ki67>20%	20	0.897	0.415	0.032
Ki67≤20%	21	1.205	0.316	

*derived from two-sided Mann-Whitney-U test.

Regarding prognostic factors and clinicopathological factors, positive correlations were identified regarding tumor size for p53 (P = 0.013) and ki67 (P = 0.018). Stage, lymphovascular invasion (LVI) and grading were each negatively correlated with p21 (P = 0.001, P = 0.001 and P = 0.0002, respectively) and positively correlated with p53 (P = 0.0005, P = 0.001 and P = 0.0002, respectively) and ki67 (P = 0.001, P = 0.0003 and P = 0.012, respectively; cf Figure 1 and Table 2).

**Table 2:** Clinicopathological features stratified by biomarker results.

Prognostic factor	N	Size (median)	Size (IQR)	≥T2+/−(n)	High grade +/−(n)	LVI +/−(n)
p21>10%	32	12	14	4/28	12/20	5/27
p21≤10%	9	24	39	6/3	7/2	6/3
p53>10%	24	14	31	10/14	16/8	11/13
p53≤10%	17	13	13	0/17	3/14	0/17
Ki67>20%	20	19	29	9/11	14/6	9/11
Ki67≤20%	21	10	14	1/20	5/16	2/19

**Figure 2:**
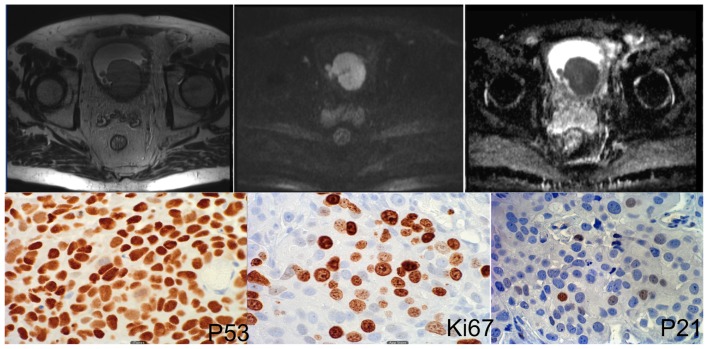
71 year old male patient. MRI images (upper row, right: T2w, middle: DWI, left: ADC map) show an intravesical mass. ADC value was measured as 0.655 *10^−3^ mm^2^/s. Lower row shows immunohistochemical stainings. Percentage of positive cells was 92% (P53), 69% (Ki67) and 1% (P21). Histopathology showed muscle invasive high-grade bladder cancer stage T2a.

**Figure 3:**
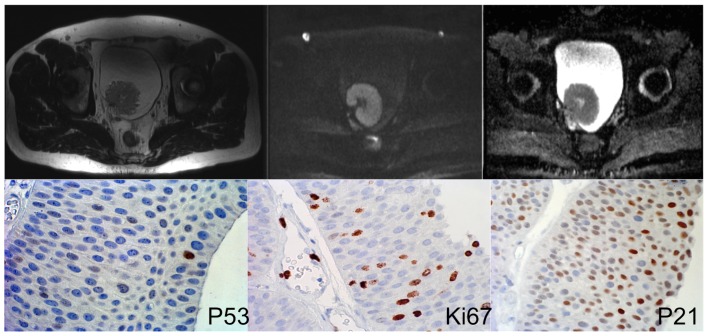
47 year old male patient. MRI images (right: T2w, middle: DWI, left: ADC map) show an intravesical mass. ADC value was measured as 1.081 *10^−3^ mm^2^/s. Lower row shows immunohistochemical stainings. Percentage of positive cells was 6% (P53), 12% (Ki67) and 71% (P21). Histopathology showed non-muscle invasive low-grade bladder cancer stage Ta.

**Figure 4:**
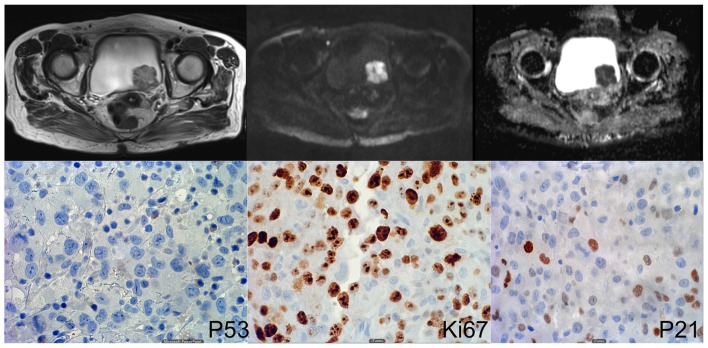
78 year old female patient. MRI images (right: T2w, middle: DWI, left: ADC map) show an intravesical mass. ADC value was measured as 0.539 *10^−3^ mm^2^/s. Lower row shows immunohistochemical stainings. Percentage of positive cells was 0% (P53), 8% (Ki67) and 23% (P21). Histopathology showed non-muscle invasive high-grade bladder cancer stage T1a.

In order to identify independent predictive factors for clinicopathological variables, multivariate logistic regression analysis was performed. ADC and p53 were both independent predictors of muscle invasion (P<0.05, respectively, cf Table 3). Both p21 and p53 were independent predictors for lymphovascular invasion; ADC and p21 were independent predictors of tumor grade. Regarding lesion size, ADC was the only independent variable selected by the regression model (P = 0.019). Detailed regression results are given in Table 3. Representative clinical examples are given in Figure 2, Figure 3 and Figure 4.

**Table 3:** Multivariate binary logistic regression models and their according area under the ROC curve (AUC) for prediction of clinicopathological bladder cancer features.

Predicted linicopathological parameter	Selected prognostic factor	Regression coefficient	Standard error	P-value	AUC (95% CI)
					0.926 (0.843–1)
<T2 vs. ≥T2*	ADC	−0.005	0.002	0.013	
	p53	0.007	0.003	0.018	
LVI^°^	p21	−0.006	0.003	0.039	0.830 (0.687–0.973)
	p53	0.005	0.003	0.070	
Grading^+^	ADC	−0.014	0.005	0.010	0.981 (0.945–1)
	p21	−0.008	0.004	0.035	
Size <30 mm vs ≥30 mm^|^	ADC	−0.004	0.002	0.019	0.774 (0.578–0.970)

Method: forward feature selection (likelihood ratios).

Nagelkerke R-squared: *0.574, ^°^:0.445, ^+^: 0.852, ^|^: 0.244.

Hosmer and Lemeshow test: *P = 0.229, ^°^P = 0.901, ^+^P = 0.225, ^|^P = 0.244.

## Discussion

The present study showed significant correlations between ADC values obtained from DW-MRI and clinicopathological prognostic criteria, specifically histological grade, tumour size and muscle invasiveness. Further, significant correlations between ADC values and the prognostic immunohistochemically derived biomarkers p53, p21 and ki67 were identified. Despite cross-correlations, ADC was one independent predictor for bladder cancer stage and grade as identified by multivariate analysis. Aggressive muscle invasive bladder cancers present with low ADC values and a high fraction of ki67 positive cells. These findings logically fit to each other: an aggressive neoplasm shows a high proliferation rate as reflected by ki67 measurements while the result of this high proliferation rate leads to an increased cellularity, decreasing the proportion of extracellular to intracellular space. ADC measurements reflect water diffusion in the extracellular space and are relatively decreased in highly proliferative tumors [15]. However, the reason why ADC values are decreased in aggressive cancers is not fully understood, as several authors in several organs have demonstrated associations between ADC values and cellularity of the tumor proliferation rate, however, these correlations are weak to moderate and thus in good agreement with our own findings [9], [16]–[18]. Further, we investigated p53 and p21: p53 is also associated with tumour stage, pathological tumour grade and lymphovascular invasion. Further studies have reported that p53 over-expression is associated with high grade and higher stage in patients with bladder cancer [19]. It has also been established that p53 is an independent factor for prediction of recurrence progression and mortality in bladder cancer [19]. Recent studies have demonstrated that a combination of cell cycle regulators such as p53, p21, p27 and cyclin e1 provides superior prognostic information as compared to these markers analyzed independently [2]. The association between ADC, ki67, p53 and p21 underlines that ADC values are associated with certain phenotypes of bladder cancer, showing lower values in muscle invasive and high-grade tumors. The reason why we should be interested in another marker of malignancy is obvious: while immunohistochemically derived prognostic markers require invasive tissue sampling and human interaction in selecting representative slides for analysis, ADC values represent the result of a noninvasive, three-dimensional and quantitative test. However, based on our preliminary results, ADC values may also have an incremental prognostic value and are not an replacement for other prognostic markers. It has been suggested in a recent review, that a combination of prognostic markers in bladder cancer may be needed to provide a complete description of the underlying tumor pathology in this heterogeneous disease [19].

We are not the first to describe associations between ADC values and clinicopathological features in bladder cancer [6], [8], [9], [20], [21]. While our results are in good agreement with these previous studies, little is known about the association between ADC values and prognostic biomarkers. To our knowledge, only Kobayashi et al. conducted a study on correlations between ADC, ki67 and clinicopathological features in bladder cancer [9]. Our results go along with this prior study, demonstrating a similar correlation coefficient between ki67 and ADC (−0.47 in our study and −0.57 in the study by Kobayashi et al.). The authors concluded that ADC values are a biomarker for bladder cancer aggressiveness [9]. Our study goes beyond this initial study, as we included further markers of the cell cycle and their associations with both ADC values and clinicopathological factors. Of note, except for lymphovascular invasion, ADC values were independently predictive of all important clinicopathological features such as grade and stage in bladder cancer.

We are obliged to mention limitations of the current study. First, the number of patients included in this study are rather low. This underlines the pilot study character of our research. It has to be stressed that the exploratory multivariate models presented in this text cannot be directly applied in clinical practice as they are not prospectively validated under the same conditions under which the underlying data were collected. However, the positive correlations between the single examined factors and the computed multivariate models have each proven statistical significance and underline the interest in further research on this topic. Further, we did not investigate retest reliability data on the variability of ADC measurements if measured on two different occasions in the same patient. The low variation of ADC measurements in different tumors within this study strongly suggests a low re-test variation. As our study was intended as an exploratory analysis to identify cross-correlations between ADC values and prognostic biomarkers with clinicopathological features. Standardization and reliability analyses are thus beyond the scope of this paper. The coefficient of variation between different readers on the same images was low.

Although we identified correlations with clinicopathological and immunohistochemical prognostic factors, the possible prediction of patient outcomes in terms of disease free and overall survival would be of primary clinical interest. However, the associations identified in our study are very suggestive of a prognostic value of ADC values for patients' outcomes.

In conclusion, ADC values are both correlated with altered proliferative activity in bladder cancer as measured by immunohistochemical biomarkers and, further, correlated with the prognostically relevant clinicopathological presentation of bladder cancer. Multivariate statistics demonstrated ADC values as an independent predictor of BCA grading, size and muscle invasion. Of the investigated immunohistochemical biomarkers, p21 and p53 were predictive of LVI and p53 independently contributed to muscle invasion and tumor grade prediction. Our findings underline the potential role of ADC values as an independent and additive diagnostic biomarker for prediction of bladder cancer biological aggressiveness and provide a basis for further studies validating the utility of these findings for clinical decision-making.
